# Family physicians’ preferences for education to support family caregivers: a sequential mixed methods study

**DOI:** 10.1186/s12875-024-02320-9

**Published:** 2024-03-07

**Authors:** Jasneet Parmar, Sara Hafeez, Tanya L’Heureux, Lesley Charles, Josephine Tite, Peter George J. Tian, Sharon Anderson

**Affiliations:** Division of Care of the Elderly, Department of Family Medicine, University of Alberta, Edmonton, Canada

**Keywords:** Primary care, Doctors, Educational needs, Primary caregivers, Informal caregivers

## Abstract

**Background:**

One in four Canadians is a family caregiver. Family caregivers (carers, care-partners) are relatives or chosen family, friends, or neighbors who provide 75 to 90% of the care for people with physical or mental illness, disabilities, or frailty living in community homes and assist with 15 to 30% of the care in congregate care. However, a recent (2022) Statistics Canada population health study reports 44% of family caregivers are distressed. Family physicians and primary care teams are well-positioned to support family caregivers; yet, family caregiver needs assessments tend to be ad hoc and their most common needs remain unmet. Research recommends training healthcare professionals to enhance their knowledge and skills to support family caregivers.

**Methods:**

The objective of this sequential mixed methods research, a survey followed by qualitative interviews, was to explore family physicians’ desire and preferences for education about supporting family caregivers. 85 family physicians completed the online survey and eight took part in the interviews. Results from the survey and interviews were compared, contrasted, and interpreted together.

**Results:**

Primary care physicians expressed a desire to be better equipped to assess and support FCGs’ needs. Even though most physicians (61%) were very/confident about addressing family caregivers’ needs, 72% were highly/interested in education to support family caregivers of their patients. Topics with the most interest were assessing family caregivers needs in an organized way, assisting family caregivers to access resources, and address system and practice barriers to support family caregivers. The overarching theme running through the interviews was physicians hope for education to help change the patient-focused culture to inclusion of FCGs. The three themes reflect physicians’ conviction about including family caregivers in patient care: We need to take care of their caregivers, Practice and system barriers thwart including family caregivers, and Practical education might help.

**Conclusions:**

This study of family physicians’ preferences for education to support family caregivers will inform the development of education about supporting family caregivers for family physicians and trainees.

**Supplementary Information:**

The online version contains supplementary material available at 10.1186/s12875-024-02320-9.

## Background

Family physicians can play a vital role in supporting family caregivers’(FCGs) health and wellbeing [1–3]. Studies in the United Kingdom [4, 5], Ireland [6], Germany [7], United States [2], and Canada [8, 9] report physicians agree they are well-positioned to support FCGs [10].They have a central role in: diagnosis and management of the patient’s condition; gaining an overall picture of the care situation; providing information, education, and advice to FCGs on managing care; offering psychosocial support to the patient and family caregiver; and signposting caregivers to services to meet their care needs and to maintain their own health [11–14]. However, few physicians or other health care professionals receive training in engaging and supporting caregivers [1, 3]. Typically, the focus is on the patient in healthcare encounters. Health providers are reluctant to involve caregivers as partners on the care team, often citing lack of time [4, 15, 16].

While FCGs regard family physicians as an important part of their support network [12, 17, 18], typically, FCGs are underrecognized and overused by health care providers. Health providers expect FCGs to carry out their care plans yet surveys of FCGs report that healthcare providers have asked less than half of FCGs about what they need to manage older adults’ care [19, 20], and less than 20% about what they need to maintain their own wellbeing [21]. In primary care, FCG needs assessments tend to be ad hoc [2, 22] with their most common needs only being partially met or remaining unmet in about 40% of cases [22–25]. In a patient-focused health system, there is a reluctance to collaborate with caregivers and an unwillingness to meaningfully involve caregivers in the care process.

Training about engaging FCGs or the FCG/patient dyad is recommended [12, 16, 26–28]; however, there has been little research on family physicians’ desire for education about family caregiving or about the topics about caregivers and caregiving they perceived apply to their practices [26, 29]. As such, the objectives of this research were to explore family physicians’ desire and preferences for education about supporting FCGs and to understand physicians’ considerations for such education.

## Methods

Qualitative interpretive description, outlined by Thorne [30] was our theoretical methodological approach. It is pragmatic approach designed to embrace the complexity and contradiction of health studies. Thorne [31] describes it as “ways of thinking that acknowledge the messiness of the everyday practice world” (p. 29).

We used a sequential mixed methods design. We began with a cross-sectional quantitative online survey to assess family physicians’ confidence in meeting FCG needs, desires for education about supporting FCGs, and preferences for educational content and delivery. Then we conducted qualitative interviews to obtain an in-depth understanding of physicians’ perceptions of the education they needed to enable them to support FCGs in primary care. The university’s Health Ethics Research Board approved the study.

### Setting and participants

This study was conducted in Alberta, Canada. Family physicians were recruited to participate in the open, convenience sample survey by advertising in provincial newsletters sent to physicians and on social media (Twitter, Linked In, Facebook, and Instagram). Our inclusion criteria included practicing family physicians. Retired physicians were excluded. The anonymous survey was delivered on Survey Monkey from April 14 to August 30, 2022. Ninety people clicked on the survey link. Of those, 85 read the ethics consent document, then all 85 provided implied informed consent by continuing to, completing the survey questions, and clicking on “submit my responses” (94.4%). Survey participants who agreed to participate in a follow-up interview (*n* = 18) were asked to provide an email address on a separate survey, which was not connected to survey data results. The research assistant emailed potential interview participants with the offer of interviews on Zoom or by telephone, and information about the interviews and implied consent. Eight family doctors agreed to participate in an individual interview. As per ethics, the interviewer reviewed the informed consent questions immediately after introductions and requested verbal consent. All participants provided verbal informed consent before the interview began.

### Data Collection

Three physicians and a PhD trained researcher designed the survey and semi-structured qualitative interview guide. The ten-question survey, delivered in English, took five minutes or less to complete. We collected socio-demographic data (age, gender, years in practice), then asked about physicians’ confidence in addressing the needs of FCGs of their patients, level of interest in completing an educational session to enhance their knowledge, skills, and approach to supporting FCGs, level of interest in ten potential topics, and preferred delivery format (asynchronous online, facilitated, time required). The ten potential topics were selected based on (1) a survey developed to understand family physicians’ beliefs about supporting family caregiver and their knowledge to support them [32]; (2) a scoping review of family physicians’ perspectives on their role in supporting FCGs [28], and (3) research on the competencies healthcare providers need to support FCGs [33]. Topics included recognizing the caregivers’ roles, communicating with caregivers, partnering with caregivers, assessing caregivers’ needs; assisting caregivers navigate health and social care systems, supporting caregivers to maintain their health, changing the culture of care to support caregivers, addressing practice barriers and personal stress, examining the caregiving environment, and understanding double-duty caregivers. We used five-item Likert-type response scales to assess confidence and interest. See Supplementary Material Table 1, Survey Questionnaire.

The team met to review the survey results and revisit the interview guide to explore physicians’ experience with FCGs, if the survey results reflected physicians’ educational needs, resources they had and needed to support FCGs, and their preferences for the format and delivery of the education. An interviewer with training in qualitative interviewing [29] conducted the interviews. We asked questions such as, “Could you speak to your experience of interacting and partnering with FCGs in your practice?” “Given the context of your practice, which educational topics would be your priority?” “What are some of your go-to resources that you use to support FCGs?” and “What would make navigation/ signposting easier for you?’ See Supplementary Material 2, Interview guide.

The interviews ranged from 19 to 45 min, an average of 28 min. The interviews were transcribed verbatim, and one author listened to the interviews, corrected any inaccuracies, and cleaned the word document of any identifying information. Field notes were completed by the interviewer. Once the interviews were transcribed, the interviewer and PhD trained research used Roulston’s [34] reflective interview guide to reflect member checking, interruptions, and ways to improve interviewing.

### Data Analysis

We analyzed survey data in Excel using descriptive statistics. We imported the qualitative data into NVivo for ease of data management, then two authors, a family physician with a Master degree in Human Ecology and research coordinator with PhD in gerontology (JP, SA) analyzed it thematically [35, 36]. Following Thorne [37] and Braun and Clarke [35, 36], our aim was to generate meaning from the data. Initially, each researcher read the text to familiarize themselves with the data, then went through the transcripts line-by-line and assigned initial codes based on their interpretation of the underlying meaning. Both regarded themes as summaries of what participants said in relation to the topic [36]. Then they collated the line-by-line codes into preliminary themes, understanding that each researcher’s codes were their interpretations of patterns of meaning across the data. After theming separately, the two team members met in person to resolve theming disagreements and identify the prominent themes to be presented to the entire team. The team then compared, contrasted, and interpreted the survey results and interview themes together.

We used the Checklist for Reporting Results of Internet E-Surveys (CHERRIES) [38]; STROBE Statement [39], Checklist of items that should be included in reports of cross-sectional studies; and Standards for Reporting Qualitative Research (SRQR) [40]. See Checklists Included in Supplementary Materials 4.

## Results

The survey sample was composed of 85 family physicians. They represented all age ranges, 59% were women, 35% identified as men, and two identified as non-binary and other. About half (47%) had been in practice for 21 or more years. See Table 1: Participant Demographics. Eight physicians participated in the qualitative interviews. One has been in practice for less than 5 years and the others for 10 or more years of experience. They practiced in urban [4], suburban [2], and rural [2] Alberta settings.

**Table 1:** Demographics

Age	Number	(%)
20 to 34	7	8%
35 to 44	24	28%
45 to 54	12	14%
55 to 64	21	25%
65 +	19	23%
Prefer not to answer	2	2%
Gender		
Man	30	35%
Woman	50	59%
Transgender	0	0%
Non-binary	1	1%
Other	1	1%
Prefer not to answer	3	3%
Years in practice		
1 to 5	14	17%
6 to 10	13	15%
11 to 15	11	13%
16 to 20	6	7%
21 years or longer	40	47%
Prefer not to answer	1	1%

### Survey results

Almost two-thirds of these physicians(61%) indicated they were confident or very confident in addressing the needs of FCGs of their patients, 27% were neutral, and 8% were not confident. Even though most were confident about addressing FCGs**’** needs, 72% were interested or highly interested in education to enhance their knowledge, skills, and approach to support FCGs of their patients. See Table 2 Confidence to Work with FCGs and Interest in Education.

**Table 2:** Physicians’ Confidence in Addressing Family Caregivers’ Needs and Interest in Education

**Level of confidence in addressing needs of family caregivers of your patients?**	**Number**	**%**
Very unconfident	1	1%
Unconfident	6	7%
Neither unconfident or confident	23	27%
Confident	42	49%
Very confident	10	12%
Prefer not to answer	3	4%
**Physician’s Level of interest in education**	**Number**	**%**
Highly uninterested	1	1%
Uninterested	7	8%
Neither uninterested or interested	15	18%
Interested	41	48%
Very interested	20	24%
Prefer not to answer	1	1%

Topics which interested physicians most were assessing FCGs’ needs in an organized way (91% interested or highly interested), assisting FCGs to access resources and overcome barriers (89%), address system and practice barriers to support FCGs and reduce your own angst (80%), and supporting the emotional and psychological needs of FCGs (78%). See Table 3: Family Physicians Preferences for Potential Topics for Education about FCGs.

**Table 3:** Family Physicians Preferences for Potential Topics for Education about FCGs

Topic	% Interested
Assess support needs of Family Caregivers in an organized way.	91%
Assist Family Caregivers to access resources and overcome barriers, including community and financial supports.	89%
Address system and practice barriers to support Family Caregivers and reduce your own angst.	80%
Learn how to support the emotional and psychological needs of Family Caregivers and meet your own.	78%
Understand the unique needs of “double-duty caregivers” who work as healthcare providers and are Family Caregivers	74%
Examine the culture and context of your care setting in supporting Family Caregivers.	72%
Recognize the roles played by Family Caregivers, their diversity and consequences of caregiving.	68%
Awareness of societal views that impact Family Caregivers (i.e., ageism, stigma, discrimination).	65%
Effective communication with Family Caregivers, and use of an empathic approach.	64%
Partner with Family Caregivers and establish collaborative relationships.	62%

Almost equal proportions of physicians preferred the education delivered asynchronously online (52%) or as a virtual facilitated session (47%). Again, just under half (46%) wanted 30-minute educational sessions and 48% preferred 60 min. See Table 4 Preferences for Education Delivery.

**Table 4:** Preferences for Educational Delivery

**Preference: Delivery of Accredited Educational Sessions**	**Number**	**%**
Pre-recorded online, self study	44	52%
Virtual facilitated sessions	40	47%
Prefer not to answer	1	1%
**Preference: Length of the Educational Sessions**	**Number**	**%**
30 min	38	45%
60 min	41	48%
2 h	3	4%
3 h	1	1%
Prefer not to answer	2	2%

### Qualitative results

We were not surprised by the topics selected for the education, but we were curious to understand why physicians were confident in their approaches to FCGs, yet still thought education was valuable. The overarching theme running through the interviews was education could help to change the patient centered medical culture to include FCGs, “*I have huge hopes we will be able to create a structure in primary care of actually allowing for some of caregiver support in a team based or medical home style of care.*” (#2) They noted not being exposed to caregiver support in medical education. In practice they encountered a system focused on immediate medical needs and cure, rather than on care, “*Mostly the journey is based on cure, not helping someone’s spouse*” #1. They wanted family physicians to recognize the caregiver’s pivotal role as a partner in care. The three themes help provide a nuanced interpretation of interview participant’s views: We need to take care of their caregivers, Practice and system barriers thwart including family caregivers, and Practical Education might help.

### Theme 1: We need to take care of their caregivers

The eight physicians participating in the interviews were convinced FCGs should be recognized and supported in primary care, *“They’re obviously an important partner in what we are trying to accomplish”* (#6), yet five thought they might be unusual because *“a lot of time they get kind of left in the clinic waiting room”* (#5) or “*FCGs are invisible in those fast-paced family clinics”* (#8). Every physician interviewed acknowledged caregivers’ crucial role in supporting their patients, the importance of listening and understanding their challenges and the complexity of caregiving, including aspects of mental health, financial challenges, and emotional support.

*I really want to keep my patients at home and that depends on supporting caregivers. I think a lot of that comes down to the family practice clinics. #8*.

When asked about their interactions with FCGs, they described acting as communicators, care coordinators, conflict mediators, and advocates for caregivers. A strong focus on listening to caregivers, understanding their challenges, acknowledging the physical and emotional toll of caregiving, and letting caregivers know they appreciated them was threaded through the interviews.

*I will be also always checking in with family and seeing how they’re doing emotionally, see how they’re doing physically, see whether they need more support from home care and more support in the home, or what questions or concerns they have. And I think that it’s a very important aspect of care, because often the caregivers are forgotten. #1*.

Six of eight physicians reported involving them in decision making. They recognized caregivers are diverse and their needs are multi-faceted, many of which are non-medical *“It is profoundly patient-centered, but fundamentally non-medical”* #2. Two of the eight physicians estimated that about 50% of FCGs’ needs were non-medical such as help with social and emotional support, home care, transportation, and respite. Four physicians (rural (*n* = 2), suburban (*n* = 1), urban (*n* = 1) noted that when caregiver support is not accessible, FCGs relied heavily on them for information and guidance. Rurality increased FCGs’ dependence on physicians. Both rural physicians noted caregiving rurally is done primarily by family because there were fewer resources, so “*they put all the weight on your shoulders as a family physician or the community nurses to help with decision making, which is pretty challenging”(#7).*

### Theme 2: Practice and system barriers thwart including family caregivers

While all the physicians interviewed appreciated FCGs and wanted to support them, this theme underlines the complexity of supporting FCGs in primary care. Practice and system barriers lie between how they thought caregivers should be supported and their ability to do so. They spoke about lack of resources, short appointment times, the need for a multidisciplinary team, silos in the healthcare system and between the health and community/ social care systems and underfunding of community services such as home care and respite that prevented asking FCGs about their needs or health or providing support.

*I think it is just the approach to medicine. And I think some of this is being dictated by a multitude of things outside of the immediate individual doctors’ control. It’s just a place where every doctor only has 10 min to help people and you’re practicing siloed so you don’t have a social worker or a nurse or a psychologist or a dietitian to provide that comprehensive aspect of care. You’re going to become very focused on the immediate things that need to be addressed and the immediate things that you can fix. #1*.

Family physicians who also practiced in long-term or palliative care reported it was easier to support caregivers with teams, *“in long-term care we have a social worker and occupational therapist onsite, there is more family support and guidance”* #2. They wanted primary care teams staffed by social workers, physiotherapists, occupational therapists, and nurses.

Signposting caregivers to services and assisting them to navigate health and community systems was a pain point in all interviews. Physicians cited difficulty knowing what services were available, keeping up resource lists, combined with the limited availability of services for caregivers. Suboptimal integration of home care and community services made it difficult for family physicians to refer FCGs to the non-medical services they thought were needed. Five physicians felt social workers were much better positioned to support caregivers’ access to non-medical needs, such as access to community, financial, or respite, or transportation services.

All physicians cited the need for better funding for home care, respite, and navigation to address caregiver stress and burnout. As one physician acerbically pointed out, *“You have to have the resources before you can recommend them, the availability of resources is not great”*(#3). They pointed out the disparities between community and institutional settings, *“Hospice is better supported. Institutions are better supported. We talk about the need for home care, how important it is. It is totally underfunded”* (#5). Physicians practicing rurally noted home care is stretched, “*yes, they need it, whether they’ll even show up is a problem”* (#3).

Half of the physicians were troubled by the support disparities dictated by the patients’ condition. Physician #1 described the dilemma of getting social work for patients with cancer and non-cancer diagnoses: *“Many family practices don’t have a social worker. If they were being followed by the cancer center, I could send them there.”* Several physicians underlined the focus on single conditions rather than on complexity,

*“Family caregivers can access dementia information, but for many people, dementia is only one problem they encounter. High profile for Alzheimers, cancer, palliative care, but what about musculoskeletal, neurologic, the severe bowel incontinence? What resources does that family get?”* #3.

Another spoke to differences age, “*I had a 21year old patient with a horrific syndrome, and I thought it was desperately unfair. This mother couldn’t work and had no access to respite.”* #2.

Six physicians spoke to the need for policy and culture changes enable them to support FCGs, for example, specifically caring for caregivers, “*More practice of patient-centered care, than talk of patient-centered care especially for caregivers”* (#5); more home care funding, *“the government gives all that money to hospital where we keep these people awaiting placement instead of giving that equivalent money to home care”* (#6); or upstream prevention or social determinants of health, *“for all the billions governments spend on healthcare, why can’t they invest in appropriate upstream places?”* (#6).

The first physician interviewed spoke to the burnout caused by systemic expectations for providers to do more,

*I’m tired. Everybody I work with is tired. And I just think that putting more and more on the backs or putting the onus of more and more responsibility on the backs of individual physicians is not the way to go forward. #1*.

Other participants also reported being fatigued by the pandemic, then by systemic changes and expectations.

### Theme 3: Practical Education might help

Despite the pessimist talk of the impact of systemic and practice barriers, all the family physicians who participated in the interviews saw the benefits of supporting FCGs and wanted others educated so they could all reap the benefits of empowered families, fewer hospital and emergency room visits, improved patient care, and a shared workload.

*Education is something that definitely could make a difference. It’s not just this is a nice to have, no this can make a difference in terms of people winding up in emergency, people winding up in hospital, people, the amount of care they get at home. Families feeling empowered to get more from the different resources that are available. #3*.

Physicians who took part in the interviews agreed that identifying FCGs’ support needs and then aiding them to access resources were their top primary care priorities. They emphasized the need for education on the challenges caregivers face and their unique needs. They thought that educating physicians on how to incorporate caregiver support into their medical practices, including approaches to address caregiver concerns, into the constraints of their clinical practices, *“Toolkits or practical ways I can assisting FCGs to access resources and overcome barriers, including community and financial supports. Easily accessible”* (#8).

Several physicians highlighted that medical education needs to move beyond the traditional focus on medical diagnosis and treatment to managing complex conditions practically, teamwork, and holistic person and family centered care. They noted skills like empathic communication, breaking bad news, and training in interdisciplinary and holistic care needed to support patient and caregivers along the treatment journey were scant in their medical education, although those competencies are included now.

*We get less training in how to break bad news or how to support someone through a cancer treatment or journey. Mostly the journey is based on cure, not how to help someone’s spouse figure out how to support them through that journey. #1*.

They emphasized the need for easily accessible, well-organized resource directories to assist caregivers without burdening physicians or primary care teams. They wanted education and resources that they could easily incorporate into their medical practices. Rather than purely educational programs, they recommended creating practical tools, such as searchable databases, to provide real-time, up-to-date information for physicians, FCGs, and patients.

*When I think about the point of care tools I use every day, it’s like a massive medical textbook that has a search engine… But it’s fast, current and very helpful…. Be accessible from a phone or a computer. #6*.

Rural physicians wanted education and resources specifically designed for rural environments. Physician #7 suggested including awareness of rural societal views that impact decision making. Overall, they wanted education to be concise and easily integrated into busy schedules. They thought incorporating both virtual and in person sessions would provide flexibility and cater to different learning styles.

## Discussion

Our empirical study describes family doctors’ preferences for education to support FCGs. Research studies are now recommending training for physicians on all aspects of caregiving [3] and 72% physicians participating in the survey were interested in education about supporting FCGs. Assessing FCGs’ needs in an organized way, assisting FCGs access resources and overcome barriers, and address system and practice barriers to support FCGs. The high proportion who want education is somewhat surprising as Badovinac and colleague’s [41] 2019 survey and interviews of nurses found little demand for education about supporting FCGs. They suggested that the demand to increase health providers involvement with FCGs was “coming “bottom up” from caregivers who want more support and “top down” from legislators” (p 10) rather than from health care providers.

The family doctors we interviewed were convinced caregivers could benefit from consistent support in primary care but observed that the health system and primary care organizational structure were barriers. These are not novel findings. Studies in many countries report physicians perceive they have a significant role in supporting FCGs. Back in 1993, the American Medical Association Council on Scientific Affairs [42] advised primary care physicians to systematically integrate FCGs into regular primary practices noting, *“primary care physicians are uniquely suited to the complex task of assessing and managing the individual needs of family caregivers*” (p.1283). Mitchell and Gaugler’s [43] 2021 synthesis of American summits and national reports reaffirms FCGs’ critical role in health care and *“strongly urged health and support service providers to systematically integrate caregivers into regular practice* (p. 150).

All the physicians we interviewed spoke practice and system barriers to provide support in day-to-day practice. Several noted that interdisciplinary teams made it easier to support caregivers in long-term and palliative care than it was to organize needed services for caregivers of people living in community homes. The lack of time and the challenge finding resources for caregivers is well documented in the research [12, 26, 44, 45]. Logically, physicians are requesting a medical home [1, 46, 47] which emphasizes team-based comprehensive, high quality primary care. Primary care should have close relationships with home care other local health services (hospitals, specialists, congregate care), and broader community social supports to addressFCG’s and the person’s receiving care non-medical needs seamlessly. Integrated health and community care should also reduce the onus for FCG to navigate systems and coordinate care, which is a significant contributor to FCG’s distress [48–50].

Physicians in this research noted primary care is under significant stress. Healthcare costs are rising globally. Primary care has been seen as a way to improve cost-efficiency by focusing on prevention, early intervention, and the management of chronic conditions in a community setting, thereby reducing the need for more expensive hospital-based care [51–54]. Family physicians’ roles have also expanded to screening for social risks such as food, housing, or income insecurity which requires time and increases costs [55, 56]. Paperwork and administration burden has increased [57]. Physicians in Canada cumulatively spend 48.8 million hours per year on administrative tasks, of which 18.5 million hours are unnecessary. Primary care work is undervalued compared to other medical specialties [47, 53, 58]. Martin and colleagues [58] point out that expenditures on primary care in the United Stakes decreased from 6.5% in 2003 to 5.4% in 2016 even though visit duration and number of health and preventative issues addressed per primary care visit has substantially increased. Bodenheimer [51, 52] in the United States and Flood and colleagues [53] in Canada argued primary care needs better funding and interdisciplinary teams to increase capacity and reduce physician burnout.

Over three quarters (80%) of the survey respondents wanted education to address system and practice barriers to support FCGs and reduce their own angst. When asked about what education might address these barriers, physicians in the interviews intimated that education could socialize family caregiver support. Education and training are recognized strategies to bolster evidence-based practices and change the culture and context of care [59, 60]. They suggested education on the unique needs and challenges faced by caregivers and how to address caregiver concerns and incorporate caregiver support efficiently within the constraints of clinical practice. They wanted practical tools that can easily be used in practice. Researchers [3, 61] and a Physician’s Association [29] highlight the need to develop practical resources and training to equip family physicians to identify, assess and support caregivers. While training family physicians to assess and support the needs of FCGs will not address systemic concerns, the health outcomes of FCGs and those they care for can be improved.

### Strengths and limitations

There are limitations to this research. The online survey sampling and recruitment through medical college and association newsletters and social media, while convenient, is subject to recruiting biased respondents. We suspect that many of these physicians are convinced that FCGs can be supported better in primary care. This bias likely overestimates the primary care physicians who might currently engage in education about supporting FCGs. However, when the healthcare systems, primary care, and family physicians are under significant pressure [62, 63], having 85 family doctors complete the survey is a reasonable sample. Participating physicians were from a single province, which also limits the generalizability of the findings. However, the findings resonate with research done in other provinces and countries.

We offered physicians brief interviews primarily about their educational preferences as we were aware of the stress and burnout that family doctors are experiencing. Longer interviews might have enabled a more in-depth understanding of the barriers and facilitators they were experiencing in supporting FCGs in their primary care practices. While this study may not be a comprehensive assessment, physicians weaved many of the difficulties they were experiencing and how they were addressing them into the context of discussing their educational preferences. The results are from one province in Canada with a publicly funded health care system and may not be generalizable. However, the qualitative findings that physicians recognize that family caregivers need support and organizational and practice barriers make caregiver support difficult are common findings in many countries.

## Conclusions

Primary care physicians noted that while they were working with diverse FCGs with diverse conditions, there were few resources, and resources are more available for some conditions to support family caregiver needs, navigation, and distress. They reported it was more difficult for them to organize needed services for FCGs of Albertans living at home than it was to support caregivers of congregate care residents. Physicians rated assessing FCGs’ support needs and then assisting them to access resources as their top concerns in their primary care practices. In conclusion, the results on family physicians’ choices for education to assist FCGs will inform the development of education for family physicians and medical students regarding supporting FCGs. It will be part of a suite of competency-based education for health and social care providers who interact with FCGs [33, 64].

### Electronic supplementary material

Below is the link to the electronic supplementary material.

Supplementary Material 1

## Data Availability

The datasets used and/or analyzed during the current study are available from the corresponding author on reasonable request.
