# Supplementary material for: Family physicians’ preferences for education to support family caregivers: a sequential mixed methods study
Source: BMC Prim Care. 2024 Mar 7;25:80. doi: 10.1186/s12875-024-02320-9 (PMC10918970; doi:10.1186/s12875-024-02320-9)
Supplement: Supplementary file 1 — Supplementary Material 1 [file 12875_2024_2320_MOESM1_ESM.pdf]

Supplementary File 1: Survey

1. What is your level of confidence in addressing the needs of Family Caregivers of your patients?

2. What is your level of interest in completing an educational session to enhance your knowledge, skills, and approach to support for Family Caregivers of your patients?

3. Please select your level of interest from the following list of potential topics for education in this area:

A. Recognize the roles played by Family Caregivers, their diversity and consequences of caregiving.

B. Effective communication with Family Caregivers, and use of an empathic approach.

C. Partner with Family Caregivers and establish collaborative relationships.

D. Assess support needs of Family Caregivers in an organized way.

E. Assist Family Caregivers to access resources and overcome barriers, including community and financial supports.

F. Learn how to support the emotional and psychological needs of Family Caregivers, and meet your own.

G. Awareness of societal views that impact Family Caregivers (i.e., ageism, stigma, discrimination).

H. Address system and practice barriers to support Family Caregivers and reduce your own angst.

I. Examine the culture and context of your care setting in supporting Family Caregivers.

J. Understand the unique needs of “double duty caregivers” who work as healthcare providers and are Family Caregivers.

4. Please indicate your preference for the following types of accredited educational sessions (please select one).

5. Please indicate your preference for the length of the educational sessions.

6. How many years have you been in practice?

7. How old are you?

8. What is your gender?

9. Do you have any additional thoughts/suggestions to share that would make an educational session on supporting Family Caregivers useful to family physicians?

10. Are you willing to participate in an individual interview or focus group discussion to inform the development of the educational sessions?

Supplementary File 2: Interview Guide

Individual Interview Semi-Structured Interview Guide

Interview Methodology: We are using Grounded Theory. We will adjust the interview guide as we learn how research participants make sense of their experiences and we make analytic sense of their meanings and actions.

Introduction:

Thank you for taking part in this interview. We realize you are busy, and we appreciate your time. We expect the interview will take about 30 minutes.

My name is [and something about yourself] The study team is led by Name from the University of Alberta. Name is a Care of the elderly physician. She has also been a family caregiver to her dad and now to her mother. The goal of this study is to develop an evidence-based workshop for family physicians on supporting family caregivers.

Overview:

Family physicians are positioned to bridge the gap between what family caregivers need and what they receive from the health care system. At this point in the research, we would like to share the results of the survey, which you have participated in and explore your perspective as we move forward to develop and deliver accredited education for family physicians.

We sent you Information about the study and the Ethics Consent form, I would like to get your verbal consent to the questions,

Do you understand that you have been asked to participate in a research study?	Yes □ No□
Have read the information above?	Yes □ No□
Do you understand the risks and benefits involved in taking part in this research study?	Yes □ No□
Do you understand that the discussions will be recorded?	Yes □ No□
Do you understand that you have an opportunity to ask questions and discuss this study?	Yes □ No□
Do you know how to contact the researchers if you want to ask questions and discuss this study?	Yes □ No□
Do you understand that you are free to withdraw from the study as outlined above, without having to give a reason and without penalty?	Yes □ No□
Do you understand the issue of confidentiality and anonymity outlined above?	Yes □ No□
Do you understand who will have access to the information you provide?	Yes □ No□

Do you have any questions?

Anonymity:

We would like to record the interview. (If on Zoom) You can turn off your camera if you would like. I am going to delete the video recording immediately after the interview. The digital audio recordings will be kept in an encrypted folder on a password-protected University of Alberta computer safely in a locked facility. They will be transcribed word for word.

May I record the discussion to facilitate its recollection? (If yes, switch on the recorder).

Despite being recorded, I would like to assure you in any reports or academic articles, you will not be identified. Any information that would allow you or your work setting to be identified will be removed from the transcriptions.

Guiding questions for participants:

The survey aimed to determine educational needs for supporting family

caregivers. Please describe your educational needs for supporting family

caregivers;

Ice-breaker question:

1. Could you speak to your experience interacting and partnering with family caregivers in your practice?

Continuing questions:

2. Have you had a chance to review the survey results? If not, let me share a summary (be ready to summarize). Do these results resonate in your experience? Why or Why not?
   Probe: Do the results reflect Family Physicians' needs for education?
3. Given the context of your practice, which educational topics would be priority? (Need the most emphasis?) Probe: Can you tell me why?
4. Do you have suggestions on how the educational sessions can be delivered?
5. What are some of your go-to resources that you use to support family caregivers? Probe: What resources would you like?

Final question:

6. We have asked you lots of questions, is there anything that you would like to tell us?

End of interview:

Thank you for participating. You have provided us with very thoughtful information. If there is anything else, you would like to tell us please email us.

Supplementary File C: Exemplar Quotes

Theme 1: Need to "Take Care of Their Family Caregivers"<br>1. Listening and Understanding Challenges: Emphasizing the importance of listening to caregivers, understanding their challenges, and providing a platform for them to express their feelings and frustrations.<br>2. Recognition of Caregiver Struggles: Acknowledging the emotional and physical toll of caregiving, including feelings of being overwhelmed and invisible.<br>3. Valuing and Appreciating Caregivers: Highlighting the importance of expressing appreciation for caregivers' efforts and acknowledging their critical role in patient care.<br>4. Awareness of Caregiver Burden: Recognizing the significant impact of caregiving on individuals' mental, emotional, and physical health.<br>5. Inclusion in Decision-Making: Involving caregivers in the decision-making process, especially in complex family dynamics or in cases of role reversal.	**Listening and understanding the challenges**<br>We spend most of our visit talking about the person, which we will be also always checking in with family and seeing how they're doing emotionally, see how they're doing physically, see whether they need more support from home care and more support in the home, or what questions or concerns that they have. And I think that that it's a very important aspect of care, because often the caregivers are forgotten. #1<br>I think the ability to provide the support. So psychological support from the doctor to the family caregiver and the patient and practical help, which often is a lot of that psychological aspect, is I don't have enough practical financial help for this scenario.#2<br>I truly believe that they do their best to try to provide whatever they can for the patients. #3<br>I guess the big thing is someone would come in and they're struggling with, with dealing with the situation. You have to listen to understand. #4<br>Oh, I think in large measure, most of the time it's just listening to their challenges and letting them talk about it. #6<br>what I do personally is to try and arrange meetings, family conferences... Guys, this is what's going on. How are you guys coping? #7<br>Most of them are struggling from what I've seen, maybe a lack of support or a lack of knowing where to find support. #8<br>Every patient is embedded in some context. You need to ask the caregiver to find out about that context. #5<br><br>**Recognition of the struggles**<br>So if you could actually utilize it or have some knowledge of how to get that easily without going through the a lot of rigmarole, which maybe is inevitable, but that's often a lot of people stress is actually how do I access something now not in three weeks time if and the respite part I mean, respite is huge. #2<br>Educating physicians about what resources are available and not letting them go off on a rant... getting them to understand that home cares are stretched beyond the limit. #3<br>People often get quite overwhelmed and depressed, to be honest. They come in because they're feeling anxious and overwhelmed and they just feel they can't do it anymore. #4

	In a lot of cases it might not be deemed acceptable or something to let others to know when they're feeling overwhelmed sometimes. #6<br>But people still get by. They get by. They just try to get on with it.#7<br>Most of what I've done is in terms of working with family. I hear their struggles, I see their struggles. #5<br>I just think it's really valuable if we can get more physicians to recognize the family caregiver, we'll be able to help our patients out better. #8<br><br>**Valuing and appreciating caregivers**<br>Well, what we find is caregivers with more resources are better able to thrive in their life #7<br>I usually feel and remember to sort of express appreciation for their help with everything. #6<br>They're obviously an important partner in what we're trying to accomplish." #6<br>You have to appreciate they are a family caregiver. They don't stand for BS, right? #5<br>I always say "Thank you for your contributions." #4<br><br>I think when we do education or when you guys are looking at that, it might be good to frame it that way, too. I mean, we're helping the caregiver, but we're also indirectly really supporting that patient by supporting the caregivers. #8<br><br>**Awareness of Caregiver Burden**<br>Learn how to support the emotional and psychological needs of family caregivers and meet your own. #2<br><br>But to I think the challenge is we know this is an issue. They have so much on their plate that we need highlight it as an issue, that we need to support them so it would make their lives easier in the long run.#4<br><br>A lot of them are really burnt out. Sometimes there's a little bit of support outside family that's helping out, but it's the caregiver doing the specific care. #8<br><br>**Inclusion in Decision-Making**<br>We're not just taking care of the patient, we're also taking care of their family or whoever their chosen family is.#1<br>I've learned the importance of making sure the caregivers are included in all of the discussions. #8

	And often the struggle is that the person, especially if it's an older person, doesn’t want to be involved in decision making. That's probably more common when it is a son or daughter making decisions for a parent. #4<br>I try to make sure, I get them involved in the treatment plan. You can't help them if you don't have any collaborative arrangement, then sometimes they won't open up to you. #5
Theme 2: “Practice and System Barriers to Supporting Family Caregivers”<br>1. Lack of Resources and Support: Highlighting the shortage of easily accessible resources and support systems for caregivers.<br>2. Time Constraints in Clinical Practice: Addressing the challenge of limited time in clinical settings to adequately support caregivers.<br>3. Challenges in Engaging Physicians: Difficulty in getting physicians to engage in new educational initiatives or to focus on caregiver support due to their busy schedules.<br><br>4. Systematic Barriers: Identifying systemic issues, such as bureaucratic hurdles and a lack of integrated care models that hinder effective caregiver support.	**Lack of resources and supports**<br>I think addressing support needs in an organized way is very important because I think often sometimes we're just kind of patching together random solutions. #1<br>The biggest challenge is knowing what's available... If you are the family care physician and you don't know and really this isn't medical, this is social stuff and there's not really a social worker available that you can chat to. It becomes very difficult to know. #2<br>I really very quickly realized that there was a gap there because my patients needed social work. I was SOL. And you have these people really complex and these you know, maybe they're applying for AISH. Maybe they need help with their taxes or maybe they need help with transportation reimbursement if they're having to drive from Lethbridge to Calgary. #1<br>But if you don't have a well-known condition, you're kind of luck. So my patients with myotonic dystrophy don't have that kind of support. #3<br>There are often times where there isn't a resource in existence, or if there was, I’m not sure of it, especially for some of the low-profile conditions. #3<br>You have to have resources before you can recommend them, right? Especially financial. #3<br>Those work with the care provider like myself... It is challenging, you know. But people still get by. #7<br>If there was a little menu that I could, access, if you're having a problem with getting them around. #4<br>I don't feel like I necessarily do a very good job on that front in terms of accessing the probably hundreds of programs that might be available or not. #6<br><br>I don't necessarily have the direct resource to give the family caregiver. #6<br>I don't really have in my head or an easy, accessible resource that would be able to share detailed website or phone numbers or this kind of thing. #6

	It's overwhelming and onerous task to imagine that I'm going to try and keep up with all the various support agencies and whatnot. #6<br>We need know what supports there are. I don't think there's any resource like that, in a rural area. So it would be a good idea if that can be developed and then maybe trialed and put in place to support the care system in the rural setting. #7<br>**Time Constraints in Clinical Practice**<br>I think some of this is being dictated by a multitude of things outside of the immediate individual doctor's control, right. How much time you have to see people and that bleeds into everything in your day. #1<br>I think sometimes we miss recognizing the role of the caregiver. We just don’t have the time. I think sometimes we forget that people come into the hospital and we spend all our time talking to the patient, but we forget to see, you know, maybe they ended up in the hospital because the caregiver is super burnt out or isn't getting enough support at home, and it isn't actually a purely medical admission. #1<br>It's the fee for service model that makes physicians less willing to give up time to do things right. #2<br>I can give them some advice and information what to encounter but I don't, you know, I can't sit there for half an hour or 45 minutes going through all the different things that could occur #3<br>And of course I would love to create resources with caregivers, but that's not the nature of our conversation, it is not possible in a busy practice. #3<br>And they tend to think that, especially in palliative care, you refer them. Okay. That means another doctor is going to take care of them. #3<br>I don’t really have time for one or two more questions. #7<br>Sometimes they don't want to open up a can of worms because of the time, right? So you’re kind of pulled two ways, like you want to help, but at the same time, you know, there's limited time and the patient's probably already quite complex. #8<br><br><br>**Challenges in Engaging Physicians: Busy Schedules**<br>Probably the most really don’t have time from their practices #4<br>I think the challenge is people know this is an issue, but they have so much on their plate. #4

	I think for physicians, it needs to be quick and easy. We just don’t have time for more. #8<br>I don't think any amount of education is necessarily going to improve my performance when we’re just so busy. We don’t have the time to use it in practice. #6<br>A family doc cannot. It's a fixed cost. And so many people not going to let the patient or caregiver unpack anything. #5<br>But to I think the challenge is people know this is an issue, but they have so much on their plate. #4<br><br>**Systemic Barriers**<br>I think, first of all, part of it is just the approach to medicine... every doctor only has 10 minutes to help people and you're practicing siloed so you don't have a social worker or a nurse or a psychologist or a dietitian to provide that comprehensive aspect of care. #1<br>It's the fee for service model that makes physicians less willing to give up time to do things right. #2<br>It's a very fragmented approach... it's a very fragmented approach... it's often difficult to get the kind of support in the community for family caregivers to be able to access information. #3<br>So, it's a very fragmented approach. If you have something like A.L.S. or muscular dystrophy, you'll get some education through the neuromuscular clinics. #3<br>It’s a challenge to break through the silos and get them the help they need. But for those who are seeing their family physicians regularly, there definitely can be some interventions earlier. The problem is that often they take only the really stressed caregivers. #8<br>You know, there's a few maybe high fliers, you know, like Alzheimer's Society or maybe Parkinson's or other kind of specific disease state kind of groups that have had higher profile that, you know, I make reference to. But the others, I would have to go searching for them. #6<br>Many needs are non-medical. You know, you need to answer the question, is it direct physician work? We need a collaborative team that provides comprehensive care in the medical home. #6<br>I'd just rather have more resources and be part of a true interdisciplinary team rather than, again, just talking about what we should do or could do. #5
Theme 3: What is Needed in Physician Education	**Education on Caregiver Needs and Challenges**

1. Education on Caregiver Needs and Challenges: Emphasizing the need for physician education on the unique needs and challenges faced by caregivers.<br>2. Resource Awareness and Utilization: Training physicians to be aware of and utilize existing caregiver resources and support programs.<br>3. Practical and Accessible Educational Tools: Developing concise, practical educational tools and programs that can be easily accessed and used by physicians.<br>4. Integrating Caregiver Support into Medical Practice: Educating physicians on how to incorporate caregiver support into their regular medical practice, including efficient approaches to address caregiver concerns within the constraints of clinical practice	I think that a lot of our training is still focused on the very hard aspects of medicine and how to find a diagnosis, how to treat it, how to work within the hospital systems that we're often trained in. But we get less training, although it's much better than it used to be in how to break bad news or how to support someone through a cancer treatment or journey. Mostly the journey is based on cure not how to help someone's spouse figure out how to support them through that journey. #1<br>There's a lot that I do that really would not be considered for medical and that you could argue, is not really my job, but although I do have a social worker on my team and they're very capable, it still is really important to get this information right off the bat to determine who should be referred to the social worker for financial concerns or for help with disability forms. Those things are important to determine. So there are visits where I spend half or even less than that talking about what you would consider standard medical things.<br>I think you have to emphasize that caregivers need different kinds of support at different times. #6<br>So if there are protocols to speak. Developed to cater specifically for rural setting... it becomes quite difficult to assist the caregivers. #7<br>I think physicians know how to assess like mental health and things like that, but they might not. It might be good to have a guide to figure out the best way to navigate, because it's a little bit different than just like mental health. #8<br>Complex care. Dementia is only one of the problems that the elderly encounter in the community that can lead them to needing a lot of extra care from the family. #3<br>What you want to do is make sure that the family caregivers have the support and psychologically and mentally and physically and whatever else they need socially. #2<br>**Resource Awareness and Utilization**<br>One of the residents for their scholarly project made an app that had all the social services in one place... putting all this information in one place, provincewide or citywide that people could access, it would be very helpful. #1<br>I think if there is a hub... if there is a hub of knowledge as to what's available and a real network of information, I think having information evenings or leaflets or some access point would be really useful. #2<br>Engagement, Yeah. How So things like the College of Family Physicians in Canada. We have the Alberta chapter, and have an annual meeting at the beginning of March every year,

	trying to tap into that either as a booth or doing a presentation . #4<br>I need to know how to help them access resources and overcome barriers, including community and financial supports. #5<br>I mean, part of the reason I do palliative care is because there are resources I can access to help. #5<br>What is available for less well known conditions. If you don't have a well-known condition, you're kind of luck. #3<br>Being honest, that the availability of resources is not great. #3<br>It's consistent across every conversation that they'd like to see... a resource pool that could be online, that could be updated yearly, bi-yearly, that could be accessible by physicians to just be like, Hey, we have problems with X, Y, Z, what should we access? #7<br>They need to be trained to include the caregiver. I think a lot of time they get kind of left in the clinic waiting room. Like I said, other people are talking just to the patient and not really getting the perspective or even talking to the caregiver. #8<br>**Practical and Accessible Educational Tools**<br>What can you give me to take back and use in practice? #4<br>Put the money into an I.T. navigation interface and which is maintained accurate and current #6<br>It's being able to quickly, easily put my fingers on what that support might be for the individual and to be able to give them that information. #6<br>It's more effective if it will be online #7<br><br>**Integrating Caregiver Support into Medical Practice**<br>How do we take that education piece and translate it to those they work with, i.e., the nurses? #1<br><br>I think the ability to provide the support in my practice. So psychological support from the doctor to the family caregiver and the patient and practical help, which often is a lot of that psychological aspect, #2<br><br>Education needs to be easy to put into practice. I want what I can use #4<br>In large measure, most of the time it's just listening to their challenges and letting them talk about it. So integrating that needs to be, how to do that in the time you have available. #6

	So how do you incorporate those family conferences, we call them family meetings into practice #7<br>Like I said, I've learned the importance of making sure the caregivers are included in all of the discussions. How do you do those conversations with patients and family caregivers #8)

Supplementary File D: Cherries Checklist

Checklist for Reporting Results of Internet E-Surveys (CHERRIES)

Item category	Explanation
Design	The online survey study involved a convenience sample. We used a sequential mixed methods design. We began with a cross-sectional quantitative online survey to assess family physicians' confidence in meeting FCG needs, desires for education about supporting FCGs, and preferences for educational content and delivery. Then we conducted qualitative interviews to obtain an in-depth understanding of physicians' perceptions of the education they needed to enable them to support FCGs in primary care. Eligibility criteria included practicing physicians in Alberta
IRB	**Approval.** The study has been approved by the University of Alberta HREB<br><br>**Informed consent.** The anonymous survey was delivered on Survey Monkey from April 14 to August 30, 2022. Ninety people clicked on the survey link. Of those, 85 read the ethics consent document, then all 85 provided implied informed consent by continuing to, completing the survey questions, and clicking on "submit my responses" (94.4%). Please see the Supplementary File for survey.<br><br>**Data protection.** No personally identifying information was collected.
Development and pre-testing	Three physicians and a PhD trained researcher designed the survey and semi-structured qualitative interview guide. The ten-question survey, delivered in English, took five minutes or less to complete. We collected socio-demographic data (age, gender, years in practice), then asked about physicians' confidence in addressing the needs of FCGs of their patients, level of interest in completing an educational session to enhance their knowledge, skills, and approach to supporting FCGs, level of interest in ten potential topics, and preferred delivery format (asynchronous online, facilitated, time required). The ten potential topics were selected based on 1) a survey developed to understand family physicians' beliefs about supporting family caregiver and their knowledge to support them (31); 2) a scoping review of family physicians' perspectives on their role in supporting FCGs (27), and 3) research on the competencies healthcare providers need to support FCGs (32). Topics included recognizing the caregivers' roles, communicating with caregivers, partnering with caregivers, assessing caregivers' needs; assisting caregivers navigate health and social care systems, supporting caregivers to maintain their health, changing the culture of care to support caregivers, addressing practice barriers and personal stress, examining the caregiving environment, and understanding double-duty caregivers. We used five-item Likert-type response scales to assess confidence and interest. See Supplementary Material Table 1, Survey Questionnaire.

Recruitment process	**Survey type.** Open online survey.<br><br>Recruitment Family physicians were recruited to participate in the open, convenience sample survey by advertising in provincial newsletters sent to physicians and on social media (Twitter, Linked In, Facebook, and Instagram). Our inclusion criteria included practicing family physicians. Retired physicians were excluded.
Survey administration	**Web/E-mail.** Participants were given a weblink<br><br>**Context.** This study was conducted by Dr. Jasneet Parmar, Professor, Department of Family Medicine, University of Alberta .<br><br>**Mandatory/voluntary.** The survey was voluntary.<br><br>**Incentives.** None.<br><br>**Time/Date.** Delivered on Survey Monkey from April 14 to August 30, 2022.<br><br>**Randomization of items or questionnaire.** N/A<br><br>**Adaptive questioning.** N/A<br><br>**Number of items.** 10 questions<br><br>**Completeness check.** Manual completeness checks were done during the data analysis phase.<br><br>**Review step.** Participants could use a Back button.
Response rates	**View rate** Ninety people clicked on the survey link.<br><br>**Participation rate** Of those, 85 read the ethics consent document, then all 85 provided implied informed consent by continuing to, completing the survey questions, and clicking on "submit my responses" (94.4%).<br><br>**Completion rate.** 85/85 with a few questions not used
Preventing multiple entries	**As per ethics, anonymous.**
Analysis	**Handling of incomplete surveys.** Completed surveys were analyzed.<br><br>**Questionnaires submitted with an atypical timestamp.** N/A<br>**Statistical correction.** N/A
